# Quantifying cereal crop movement through hemispherical video analysis of agricultural plots

**DOI:** 10.1186/s13007-019-0437-5

**Published:** 2019-05-24

**Authors:** Alexander Q. Susko, Peter Marchetto, D. Jo Heuschele, Kevin P. Smith

**Affiliations:** 1 Department of Agronomy and Plant Genetics, University of Minnesota, 411 Borlaug Hall, 1991 Upper Buford Circle, St. Paul, MN 55108 USA; 2 Department of Bioproducts and Biosystems Engineering, University of Minnesota, 1390 Eckles Ave., St. Paul, MN 55108 USA

**Keywords:** High throughput phenotyping, Image analysis, Lodging, 360 Camera, Oat, Wheat, Barley

## Abstract

**Background:**

Violent movement of crop stems can lead to failure under high winds. Known as lodging, this phenomenon is particularly detrimental to cool-season cereals such as oat, barley, and wheat; contributing to yield and economic losses. Phenotyping the movement of cereal crops in real-time could aid in the breeding and selecting of lodging resistant cereals. Since no methods exist to quantify dynamic, real time plant responses in an agricultural setting, we devised a video analysis protocol to quantify mean frequency and amplitude of plant movement for a 360° field of view camera system.

**Results:**

We present both the image analysis method for identifying predefined regions of a 2D field design as they appear on 360° field of view video, as well as a signal processing pipeline to quantify movement from time varying color signals from plot canopies within these predefined field regions. We detected significant differences in the natural frequency and amplitude of plant movement from video of 16 cereal cultivars planted in a randomized complete block design on five different windy days. Natural frequencies quantified by this method averaged 1.37 Hz, while over 2.5-fold differences in amplitude within similar frequency ranges were detected across the 16 cereal cultivars.

**Conclusions:**

This method is sensitive enough to systematically differentiate small frequency and amplitude differences in cultivar movement, and shows promise for investigating the physiological basis for differences in cereal movement and lodging resistance. The relative accuracy of the plot demarcation protocol suggests it could be used for other high-throughput phenotyping applications that require both high image resolution and a large field of view.

**Electronic supplementary material:**

The online version of this article (10.1186/s13007-019-0437-5) contains supplementary material, which is available to authorized users.

## Background

Prior to the failure (lodging) of plant stems under wind stress, an entire plant experiences movement. This movement occurs in plants of all sizes and taxa; it is dependent on plant structural traits and wind conditions, which together govern the failure velocity at which a plant stem will fail in the wind [1, 2]. Increasing stem resistance to wind is of critical interest to breeders of cereal crops such as oat, barley, and wheat, where development of lodging resistant varieties is a continuous challenge. Lodging in cereals is a major contributor to yield loss, which can be as severe as 31–37% when stems are lodged at a 45° angle or greater [3, 4]. While most strategies for improving lodging resistance focus on scoring lodging after a storm or measuring physiological parameters such as stem strength, less is known about how plants interact with the wind in real time, nor do methods exist to systematically quantify plant movement in agronomic or breeding experiments.

Plant movement has been studied theoretically from the scale of individual plants to entire canopies [1, 2]. At a basic glance, plant movement is governed by the spring constant (stiffness, K) of the stem [5]. In the simplest representation, the natural frequency (*ω*_*n*_) at which a plant stem moves is governed by the ratio of stem stiffness to mass per unit length (*m*_*p*_) of the stem [5]:

1 $$\omega_{n} = \sqrt {\frac{K}{{m_{p} }}}$$

From Eq. 1, an increase in the stiffness of the stem, holding *m*_*p*_ constant, will increase the natural frequency of the plant oscillations (movement). If the intermittent gusts across a cereal canopy encounter plants whose *ω*_*n*_ matches that of the gusts, the movement of the plant will not dissipate and will likely be amplified. If the vibration is sustained long enough to cross a failure threshold, the plants will lodge. However, additional physical parameters and the wind conditions all interact in reality to make plant movement a function of various physical properties at any given time point. These can include the structural (*ζ*_*s*_) and aerodynamic (*ζ*_*a*_) damping coefficients of the plant, stem acceleration ($\ddot{\theta }$), stem velocity ($\dot{\theta }$), stem angle (*θ*), and the wind moment (force of wind multiplied by area exposed, *M*_*w*_). These values can be used to estimate the *ω*_*n*_ at a given moment by the equation [5]:

2 $$\omega_{n} = \sqrt {\frac{{K\left( {\ddot{\theta } + 2\omega_{n} \left[ {\upzeta_{s} +\upzeta_{a} } \right]\dot{\theta } + \omega_{n}^{2} \theta } \right)}}{{M_{w} }}}$$

An increase in plant natural frequency should increase the wind velocity required to induce failure and result in greater lodging resistance, because stem oscillations would dissipate after being excited by gusts that occur at a lesser frequency [6]. Realistically however, any change in physiology (i.e. mass per unit length, structural loading) or environment (i.e. wind moment) will affect the resonant frequency and thereby change the failure velocity [6, 7] (Eqs. 1, 2). These expectations have been used to derive detailed equations relating plant movement and aerodynamic forces, which consider the natural frequency of plant stems as a component along with other physiological traits to model the failure of plants under wind stress [1, 7, 8]. In agronomic crops, plant movement has been factored into models that describe theoretical failure velocities in wheat, barley, and maize for given certain physiological parameters [6, 9, 10]. However, the physiological factors incorporated into these models are not easy to quantify quickly, nor is there a large scale validation method for models of plant movement that could be employed across variable germplasm. Thus, quantifying the phenomenon of plant movement relatively quickly that is scalable to large field experiments could lead to optimal phenotypic combinations for breeders to target that increase the wind resistance of cereal crops.

Empirical studies on plant-wind interaction employ a variety of technologies to quantify movement in plant systems primarily developing or validating theoretical models on a small scale. A large portion of experimental research on plant movement has focused on understanding tree sway to minimize timber losses from storms [2]. The earliest research on tree movement involved using a stopwatch to calculate a tree sway period. The study reported that as tree height increased, tree sway frequency decreased [11]. Digital data on plant movement from a micrologger (accelerometer, gyrometer) data acquisition system further allowed theoretical assumptions of plant movement to be refined on the individual plant level in maize by the late 1980s [5]. The advent of digital video cameras have enabled new quantifications of plant movement, such as adapting the technique of Particle Image Velocimetry (PIV) to light reflected off of an alfalfa canopy to map turbulent air flows over the crop canopy [12]. Interactions between individual plants have also been quantified through video analysis, specifically by tracking changes in color within a given region of a video as stems oscillate and collide with each one another [13]. Color changes within defined regions of digital video, analyzed as time-varying signals, can thus be a non-invasive measure of plant movement.

Treating plant natural frequency (*ω*_*n*_) and the amount of movement (amplitude) at that frequency as plant phenotypes could enable genetic studies and breeding based on a crops’s real-time response to field wind stress. Implementing a method to quantify phenotypes of plant movement at high throughput in experimental designs such as the randomized complete block (RCB) pose another use for relating physiology, movement, and lodging: to better optimize physical trait combinations that are desirable in theory but in practice will require high statistical power to validate under variable field environments. While no systematic approaches exist for quantifying plant movement for purposes of improving crop lodging resistance from video, many studies have devised methods to quantify crop lodging from image and spectral data. Recent lodging phenotyping efforts include analyzing lodging in the visible spectrum from unmanned aerial vehicle (UAV) images in wheat, rice, and maize [14–16]. Additionally, lodging has been quantified in the field using merged thermal and infrared images, as well as through plant height differences detected through LIDAR [17, 18]. UAV systems are not ideal for capturing video data, especially when data on plant movement is desired under high wind or stormy conditions. Ground based, automatable camera systems are more amenable to capturing video under inclement weather, and 360° field of view video cameras are capable of imaging a full hemisphere per frame [19]. The use of a 360 video camera alleviates the need for multiple cameras to capture replicated plots and for synchronization of separate videos at exact moments in time, while remaining close enough to the canopy level to capture small differences in movement. Though hemispherical video can capture a large field of view, the analysis and demarcation of individual plots is more trigonometrically rigorous and requires novel analysis protocols. For purposes of measuring plant movement, the two can be combined in a way that any field design can serve as input to guide image analysis and data organization.

In this paper, we develop novel video analyses and demonstrate that they can be used to quantify plant movement as a phenotype in the field from a stationary, 360° field of view camera system for purposes of understanding cereal crop lodging resistance under field wind stress. The context of this method focuses on individual plant rows as opposed to canopy dynamics of uniform fields, with the idea that this method could be generalized to an applied breeding scheme. The method presented quantifies the natural frequency (*ω*_*n*_) of movement and amplitude of single, homogenous rows of cereal cultivars without mechanical interference. Our method accomplishes this primary objective by (1) identifying plots from a predefined field design to systematize data analysis, (2) employing signal processing techniques to analyze plant movement as changes in the RGB color space, and (3) demonstrating the method’s capabilities by quantifying plant movement in the field for 16 cereal varieties planted in a randomized complete block (RCB) design.

## Results

### Automatic demarcation of single rows from hemispherical video

**Fig. 1:**
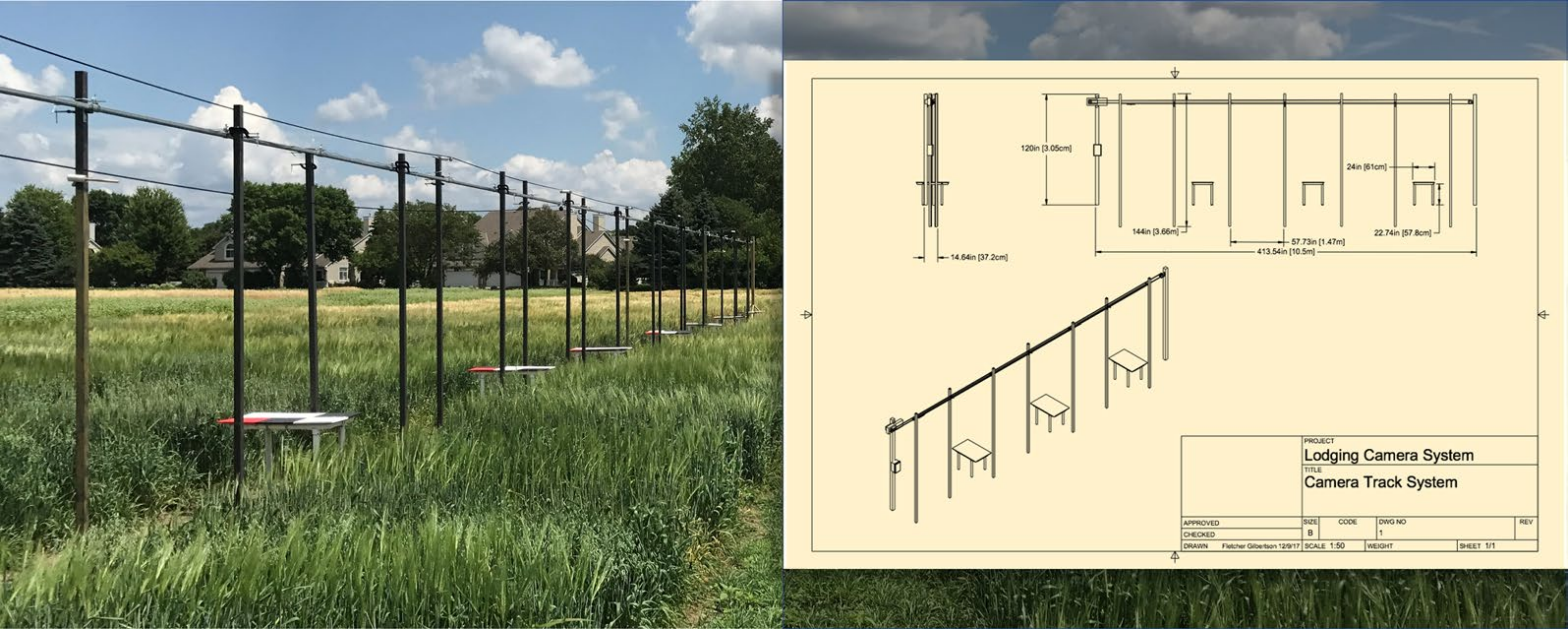
Camera track system (figure from [9]). The automatable camera track system that to which the 360 camera was mounted. Reproduced with permission from the journal

An automated camera track system [19] with a 360fly (360fly, Inc.) camera mounted 3 m off the ground, allowed us to capture hemispherical videos of 98 single row plots within a RCB design planted at four different planting dates (Fig. 1) (Table 1). The RCB design contained four cereal crop species with 16 cultivars in total (Table 1).

**Table 1:** Cultivars used in the lodging field design

Latin name	Crop	Cultivars
*Avena sativa* L.	Oat	‘Gopher’, IL078721, ND021052, ‘Reins’
*Hordeum vulgare* L.	2-Row Barley	‘AC Metcalf’, ‘Conlon’, ‘ND Genesis’, ‘Pinnacle’
*Hordeum vulgare* L.	6-Row Barley	‘Celebration’, ‘Quest’, ‘Stellar’, ‘Tradition’
*Triticum aestivum* L.	Wheat	‘Linkert’, MN113946, ‘Rollag’, ‘Shelly’

**Fig. 2:**
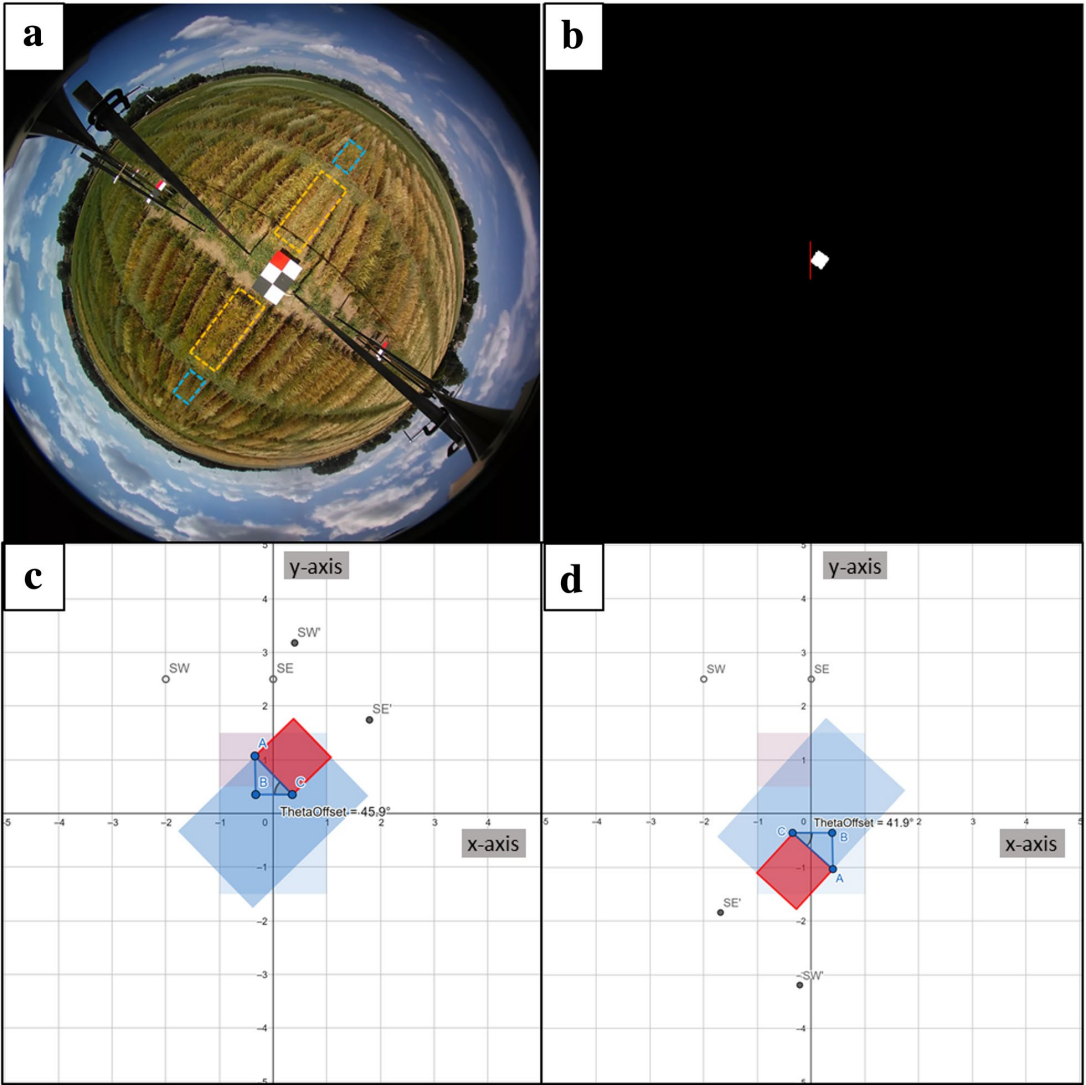
Rotation of field coordinates. The camera orientation is determined from still frames (**a**). The sample still frame shows the position of the inner (orange dashed rectangles) and outer (blue dashed rectangles) replicates in view with respect to the camera track (**a**). The red square on the panel is masked out from the rest of the image, and the leftmost corner is identified with the color values ± 40 pixels from this corner used to determine which way the panel is facing (**b**). Once the direction and degree in this direction that the image is rotated is quantified (*Θ*_*off*_), the field design coordinates are rotated in that direction such as 45.9° toward the Northeast (**c**) or 41.9° toward the southwest (**d**)

The camera track system was specifically designed for quantifying lodging and plant movement under field wind conditions, with open source design plans [19]. Four replicates were in view of the camera track (two inner and two outer replicates), with 2-minute videos taken at each of the four planting dates on five different windy days in July 2017 for a total of 20 videos capturing cereal movement (Table 2; Fig. 2a).

**Table 2:** Video date and wind speeds

Video date	Average wind speed (kph)	Gust (kph)	Direction (°)
10 Jul 2017	10.3	14.6	357
11 Jul 2017	6.7	10.3	112
12 Jul 2017	14	18.6	268
13 Jul 2017	10.2	12.9	345
17 Jul 2017	17.8	26.4	164

Average wind speed, gust, and direction at each video date

**Fig. 3:**
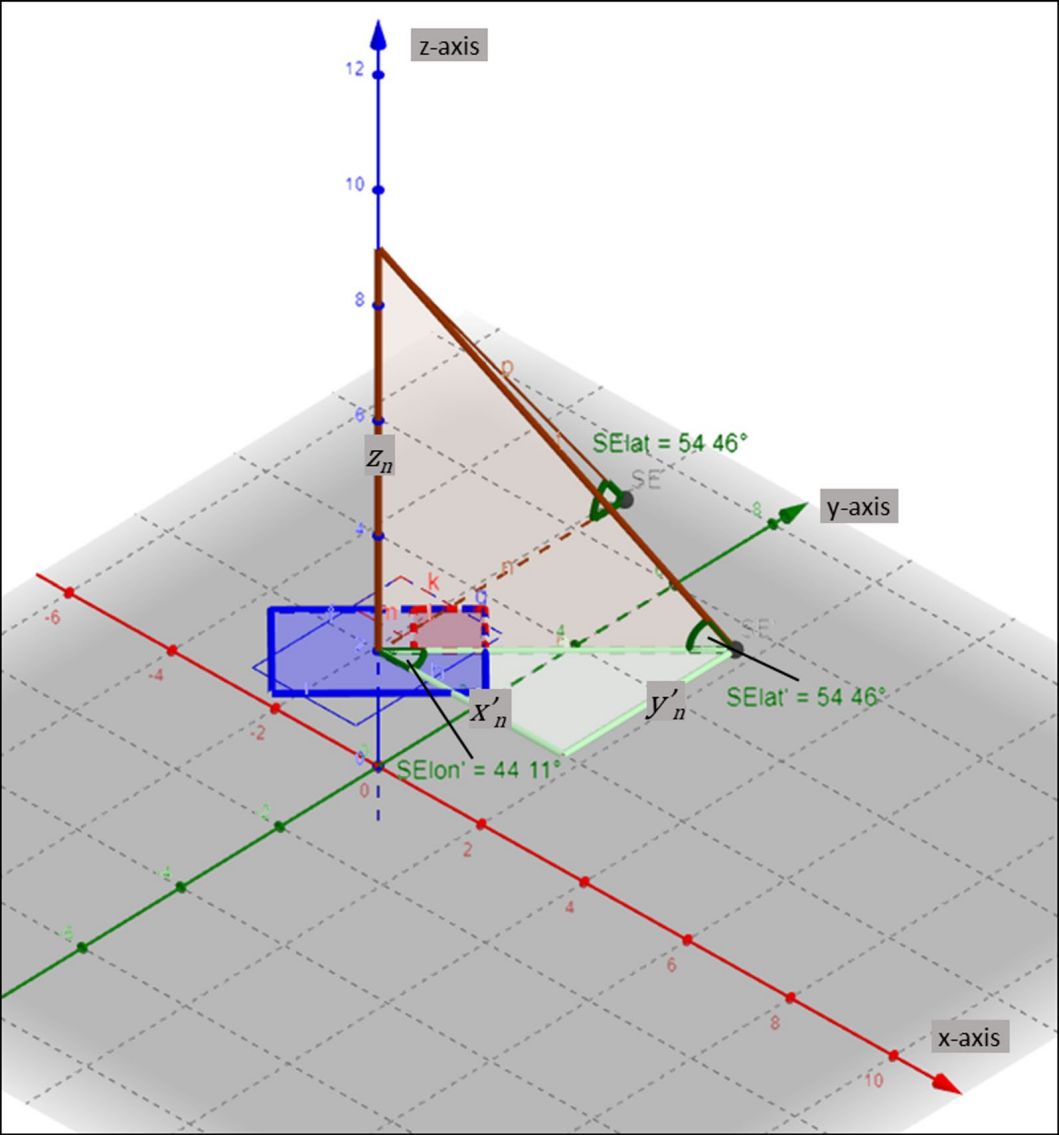
Spherical transformation via triangulation. After accounting for camera rotation using *Θ*_*off*_, distances to the rotated point (*x’*_*n*_, *y’*_*n*_) are used to calculate the longitude, and along with height (*z*_*n*_) the latitude of a point of interest transformed on to a spherical plane

**Fig. 4:**
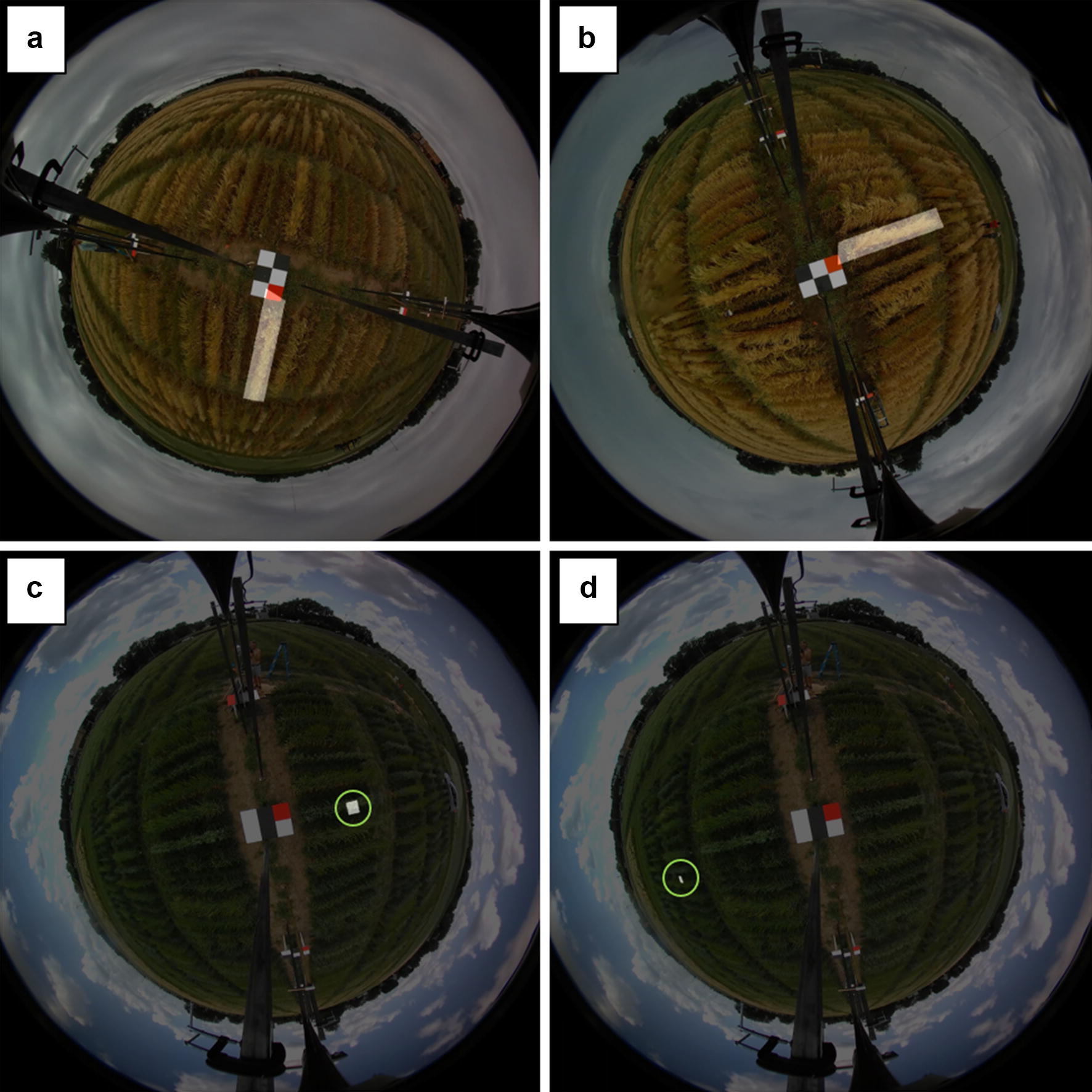
Cropped images showing automated plot identification results. Two still frames from different video dates (**a**, **b**) of the same oat plot (‘Gopher’) demarcated in two different orientations. Two still frames from the same video date below showing the 929 cm^2^ demarcation for the analysis of plant movement. ‘ND021052’ Oat as an inner rep (**c**—green circle highlight) and ‘Quest’ 6-row Barley as an outer rep (**d**—green circle highlight)

By centering the camera over a series of unique panels at each planting date, we calculated the orientation of the field with respect to the camera axes in a given video and subsequently rotated the input field design at that panel accordingly (Fig. 2a–d). The red square on each panel also served to normalize red color values in the RGB frames for comparing color values at a given plot under different lighting conditions. Following this rotation, we transformed the rotated 2D field coordinates into 3D spherical coordinates (Fig. 3). The latitude for a given point was used to obtain the number of pixels in the image out to that point, with the longitude used to calculate the number of indexed rows, columns in the image matrix using a law-of-sines relationship. This created a series of four transformed points for each plot, expressed in indexed pixels, that could then be used in cropping functions. We used the plotfinder matlab script (Additional file 1) to automatically generate both cropped images and cropping functions that used these pixel products of the field design transformation to illustrate demarcations of an entire cultivar row and 929 cm^2^ regions for quantifying plant movement (Fig. 4a–d). Visualized on the first still frame, the cropped image of ‘Gopher’ oat is seen in two different videos whose rotation in relation to the field panel differs (Fig. 4a, b). The 929 cm^2^ regions at canopy height for plant movement analysis is shown in a replicate adjacent to the camera track (inner reps) (Figs. 2a, 4c) and not adjacent to the camera track (outer reps) (Figs. 2a, 4d). Regions of the same size in the field become noticeably smaller in outer replicates when demarcated in the 360fly still frames (Fig. 4d). Along with a cropped image displaying the polygon region of interest, the cropping function generated using Additional file 1 can then be used to crop 929 cm^2^ regions at each plot and analyze the color components of these plot segments over all video frames to quantify plant movement.

### Accuracy of the automatic plot demarcation

We assessed the accuracy of the plot demarcation method over the 5 video dates by comparing manual rotation angle (*Θ*_*off*_) calculation and cropping with the automatic *Θ*_*off*_ calculation and cropping functions generated by the plotfinder script. The small *Θ*_*off*_ differences (averaging between 0.66° and 2.42°) between automatic and manual estimation propagated larger transformation errors for outer replicates compared to the inner replicates adjacent to the camera track (Table 3). These differences in combined x, y plot coordinate values averaged 1.78 cm at the inner reps and 8.74 cm at the far reps due to *Θ*_*off*_ calculation error propagation (Table 3).

**Table 3:** Errors in plot demarcation induced from manual versus automatic *Θ*_*off*_ estimates

	10 Jul 2017	11 Jul 2017	12 Jul 2017	13 Jul 2017	17 Jul 2017
Avg. angle difference (°)	1.64	2.42	0.66	1.19	1.13
Inner reps (cm)	2.89	3.22	1.34	1.12	0.33
Outer reps (cm)	12.83	16.07	5.51	6.78	2.92

Average *Θ*_*off*_ differences and subsequent error propagation (in cm) for combined x and y components of the field coordinates and grouped by replicate position at each video date. Manual versus automatic (plotfinder) estimations of *Θ*_*off*_ were used and averaged across the four panels for each video date

As another metric of the plot demarcation accuracy, we measured how color signals of plant movement were affected by comparing the average normalized red value from the hand demarcated and plotfinder demarcated regions over all frames in the videos. For the inner replicates, the correlation at each video date across all planting dates and crop cultivars was significantly higher than in the outer replicates (Table 4). On the windiest day with the most plant movement (17 Jul 2017), the correlations between normalized red color values were 0.92 for inner replicates, and 0.76 for outer replicates (Table 4). Likewise on the least windy day (11 Jul 2017), the correlations between normalized red color values were lower overall (r = 0.83 for inner replicates, 0.67 for outer replicates) (Table 4).

**Table 4:** Average correlation of normalized red color values over frames by video date

Position	10 Jul 2017	11 Jul 2017	12 Jul 2017	13 Jul 2017	17 Jul 2017
Inner reps	0.83 a	0.83 a	0.87 a	0.77 a	0.92 a
Outer reps	0.69 b	0.67 b	0.77 b	0.62 b	0.76 b

Correlation coefficients (r) of normalized red values over frames between manual and automatic (plotfinder) demarcation methods, averaged over all plots, and grouped by replicate position at each video date. Unique letters indicate significant differences between mean correlation coefficients within a video date at alpha = 0.05

### Processing signals of plant row movement

**Fig. 5:**
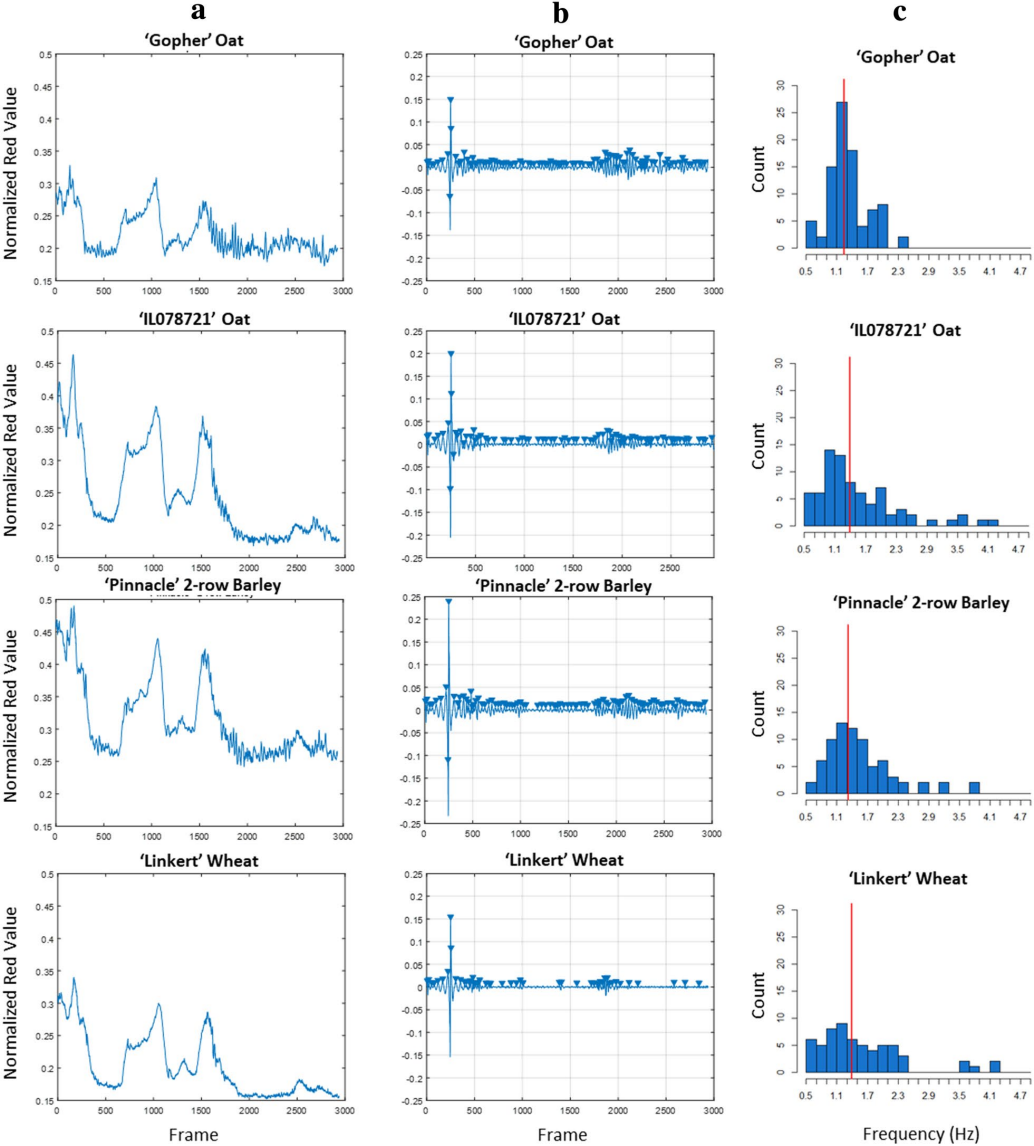
Signal processing visual. The red color value for each frame is normalized to the red color value for the red square on the panel, generating a waveform of both plant movement and outdoor lighting conditions over the video (Column **a**). The waveforms in column **a** are then bandpassed, centering the signal at 0 and removing the low frequency (< 0.5 Hz) components of the signal (Column **b**). Peaks within the bandpassed signals are identified with a minimum prominence of 0.005 Normalized Red Value, with the distance between each peak (in frames) calculated and divided by the frame rate to estimate the natural frequency (*ω*_*n*_, in Hz) to the next peak. The mean of the distribution of these frequencies constitutes the *ω*_*n*_ for the plot (Column **c**—red lines)

With the plotfinder method enabling the systematic cropping and color quantification of 929 cm^2^ plot canopy regions over each frame in a video, we devised a signal processing pipeline to quantify plant row movement from time-varying changes in color across plot canopies. We visualize a sample of plant movement waveforms by plotting the normalized red color values of four cereal cultivars taken at the panel denoting the 25 Apr. 2017 planting date on 10 Jul 2017 (Fig. 5a). The normalized red color values of the 929 cm^2^ regions over all frames in a video revealed color changes at two major scales. The first scale was low frequency (< 0.5 Hz), high amplitude color changes detectable due to changes in sunlight exposure from passing clouds during a single video, which substantially altered the red color values of the plots relative to the red panel. This is evidenced by the shared low frequency, high amplitude wave movements across all plots shown (Fig. 5a). The second color change within each of these waveforms, occurring at higher frequency (1–2 Hz) and lower amplitude, can be seen that represent the plant swaying back and forth, thereby changing the mean normalized red value within the demarcated 929 cm^2^ region (Fig. 5a). Digital bandpass filtering was necessary to remove low frequency color changes (< 0.5 Hz) due to sunlight changes and high frequency waves (> 4.9 Hz) due to camera movements and other noise, leaving only the signal of the individual row movement (Fig. 5b). The filtered signals of normalized red values are thus centered at 0 and have distinguishable peaks, which are used to calculate the mean natural frequency (*ω*_*n*_) for the bandpassed peak distribution (Fig. 5c). The movement trends of the plotted waveforms in Fig. 5a correspond to the behavior of the cultivars in the supplemental video (Additional file 2), such as low *ω*_*n*_, high amplitude movement (‘Gopher’ Oat, ‘Pinnacle' 2-row Barley) and high *ω*_*n*_, low amplitude movement (‘Linkert’ Wheat).

### Analysis of plant row movement signals

We finally used the peak detection method on the filtered signals of plant movement to generate a distribution of peaks for each plot per video date (Fig. 5c). The mean natural frequency (*ω*_*n*_) obtained from these distributions, modeled as a response in Eq. 10 with appropriate F-tests (see Methods), revealed differences among cultivars and planting dates that were significant in a ANOVA of the linear model on data from the five video dates (Table 5). The position effect of replicates nested within planting dates was significant at all video dates in explaining variation in mean *ω*_*n*_ (Table 5). The mean *ω*_*n*_ values across all cultivars was 1.37 Hz, with significant differences detected among cultivar means according to an LSD test on two of the five video dates (Table 6). Grouped in Table 6 by crop, wheat cultivars on average had higher mean *ω*_*n*_ values than the other cereals across the five video dates. Different planting dates also possessed significantly different mean *ω*_*n*_ values, though their rank was not universally consistent across the five video dates (Table 7). Notably, the windiest date (17 Jul 2017) contained almost universally lower observations of *ω*_*n*_ across cultivars and planting dates (Table 6).

**Table 5:** ANOVA *P* value results for mean by *ω*_*n*_ by video date

	10 Jul 2017	11 Jul 2017	12 Jul 2017	13 Jul 2017	17 Jul 2017
Cultivar	< 0.001*	0.011*	0.002*	0.030*	< 0.001*
PlantingDate	0.211	0.100	0.145	0.055	0.039*
Pos (PlantingDate)	< 0.001*	< 0.001*	< 0.001*	< 0.001*	0.008*
Cultivar * PlantingDate	0.405	0.369	0.441	0.372	0.448
Cultivar * Pos (PlantingDate)	0.712	0.222	0.964	0.023*	0.989

*Significant at alpha = 0.05

**Table 6:** Mean separations for mean *ω*_*n*_ (Hz) between cultivars by video date

Cultivar	10 Jul 2017	11 Jul 2017	12 Jul 2017	13 Jul 2017	17 Jul 2017
‘AC Metcalf’ 2-row	1.41ab	1.46a	1.48a	1.25bc	1.25a
‘Conlon’ 2-row	1.27b	1.46a	1.47a	1.33abc	1.26a
‘ND Genesis’ 2-row	1.51ab	1.53a	1.50a	1.44ab	1.16a
‘Pinnacle’ 2-row	1.43ab	1.41a	1.47a	1.28abc	1.11a
‘Celebration’ 6-row	1.22b	1.48a	1.54a	1.46ab	1.14a
‘Quest’ 6-row	1.37ab	1.43a	1.43a	1.30abc	1.40a
‘Stellar’ 6-row	1.45ab	1.28a	1.54a	1.32abc	1.08a
‘Tradition’ 6-row	1.45ab	1.31a	1.32a	1.28abc	1.15a
‘Gopher’ oat	1.31ab	1.29a	1.40a	1.18c	1.03a
‘IL078721’ oat	1.55ab	1.46a	1.50a	1.41abc	1.06a
ND021052 oat	1.59a	1.36a	1.62a	1.21bc	1.15a
‘Reins’ oat	1.38ab	1.20a	1.34a	1.35abc	1.06a
‘Linkert’ wheat	1.60a	1.41a	1.48a	1.43ab	1.07a
MN113946 wheat	1.57ab	1.55a	1.56a	1.35abc	1.39a
‘Rollag’ wheat	1.39ab	1.52a	1.41a	1.64a	1.48a
‘Shelly’ wheat	1.59a	1.55a	1.60a	1.52a	1.38a

Unique letters indicate significant differences between means within a video date at alpha = 0.05

**Table 7:** Mean separations for *ω*_*n*_ (Hz) between planting dates

Planting date	10 Jul 2017	11 Jul 2017	12 Jul 2017	13 Jul 2017	17 Jul 2017
25 Apr 2017	1.37bc	1.52a	1.44b	1.70a	0.85d
5 May 2017	1.47b	1.41b	1.47b	1.19c	1.01c
15 May 2017	1.62a	1.15c	1.27c	1.06d	1.26b
26 May 2017	1.30c	1.59a	1.71a	1.47b	1.53a

Unique letters indicate significant differences between means within a video date at alpha = 0.05

In addition to analyzing variation in mean frequency, we analyzed the variation in the amount of row movement (amplitude) within a 0.2 Hz frequency interval (bin) between 1.1 and 1.3 Hz. The amplitude within this frequency bin encompassed many mean natural frequencies of the cultivars in this study varied significantly among cultivars and planting dates across data from the five video dates. When modeled as a response in Eq. 10 and analyzed with an ANOVA, the 1.1–1.3 frequency bin had significant cultivar, planting date, and planting date x cultivar interaction effects (Table 8). The position effect of replicates nested within planting dates was insignificant across four of five video dates on the variation in total amplitude (Table 8). Significant differences in mean amplitude among cultivars were detected at one of the five video dates according to an LSD test (Table 9). Cultivars with a lower *ω*_*n*_ generally had more movement in the 1.1–1.3 Hz interval as measured by the sum of the red area between peaks, while those with a higher *ω*_*n*_ had less movement (Table 9). Near-universal increases in total amplitude were observed for the windiest video date (17 Jul 2019) compared to the other video dates among the cultivars (Table 9). Finally, significant differences in the total amplitude of plant movement among planting dates were detected at each video date (Table 10).

**Table 8:** ANOVA results for the total amplitude of movement (1.1–1.3 Hz bin) by video date

	10 Jul 2017	11 Jul 2017	12 Jul 2017	13 Jul 2017	17 Jul 2017
Cultivar	0.003*	< 0.001*	0.027*	0.026*	0.005*
PlantingDate	0.021*	0.055	0.277	0.052	0.017*
Pos (PlantingDate)	0.587	0.022*	0.097	0.405	0.293
Cultivar * PlantingDate	0.018*	0.050*	0.221	0.010*	0.052
Cultivar * Pos (PlantingDate)	0.714	0.514	0.342	0.159	0.511

*Significant at alpha = 0.05

**Table 9:** Cultivar differences for the total amplitude of movement (1.1–1.3 Hz bin) by video date

Cultivar	10 Jul 2017	11 Jul 2017	12 Jul 2017	13 Jul 2017	17 Jul 2017
‘AC Metcalf’ 2-row	1.59a	2.44abc	1.78a	1.46a	3.90a
‘Conlon’ 2-row	1.29a	2.11bc	2.28a	1.84a	2.18a
‘ND Genesis’ 2-row	1.88a	2.20abc	1.72a	1.37a	2.46a
‘Pinnacle’ 2-row	1.17a	2.39abc	1.75a	1.74a	3.22a
‘Celebration’ 6-row	2.39a	1.48c	1.51a	1.48a	1.56a
‘Quest’ 6-row	1.50a	3.01ab	2.24a	2.36a	3.94a
‘Stellar’ 6-row	0.88a	2.23abc	1.06a	1.16a	2.30a
‘Tradition’ 6-row	1.13a	1.91c	1.96a	2.09a	3.13a
‘Gopher’ oat	2.16a	3.12a	2.37a	2.67a	3.02a
‘IL078721’ oat	1.06a	1.48c	0.65a	0.96a	0.85a
ND021052 oat	1.19a	1.58c	1.06a	1.63a	2.35a
‘Reins’ oat	1.61a	1.53c	1.45a	1.24a	1.60a
‘Linkert’ wheat	1.29a	1.43c	1.19a	1.37a	1.41a
MN113946 wheat	0.85a	2.26abc	1.18a	1.60a	2.32a
‘Rollag’ wheat	1.24a	1.90c	1.37a	0.99a	2.18a
‘Shelly’ wheat	1.39a	1.97c	1.71a	2.13a	1.27a

Total amplitude is expressed in percent normalized red color units. Unique letters indicate significant differences between means within a video date at alpha = 0.05

**Table 10:** Mean separations for the total amplitude of movement between planting dates

Planting date	10 Jul 2017	11 Jul 2017	12 Jul 2017	13 Jul 2017	17 Jul 2017
25 Apr 2017	1.91a	1.91bc	1.80a	1.21b	0.83c
5 May 2017	1.43a	2.10b	1.54a	1.89a	3.87a
15 May 2017	0.89b	2.70a	1.82a	2.04a	2.95a
26 May 2017	1.63a	1.63c	1.43a	1.61ab	1.96b

Total amplitude is expressed in percent normalized red color units. Unique letters indicate significant differences between means within a video date at alpha = 0.05

## Discussion

Measurements of cereal crop lodging, while increasingly possible at high throughput through UAVs and image analysis, cannot capture the dynamic response that a cereal crop experiences during high winds or storms. Treating movement as a response variable could improve a breeder’s understanding of cereal wind resistance by relating cereal physiology to repeated measures of plant movement under different wind conditions and final lodging outcomes. There are obvious challenges to phenotyping plant movement on a large scale agricultural experiment using mechanical methods. Manual vibration and timing tests would prove laborious on the scale of common field experimental designs, while mechanical sensors on tens or hundreds of replicates would present data synchronization challenges for large field experiments. Thus a video analysis method using a colorimetric, signal processing approach to quantify movement offers a non-invasive measure of this dynamic phenotype that is better scaled to agricultural experiments. The desired capabilities of a scalable phenotyping method for plant movement are twofold: the method must be systematic enough to eliminate sources of human or equipment measurement errors under the intermittent winds that excite plant movement, while providing enough data of replicated varieties to elucidate trends in movement that require increased statistical power to discern in the field. The pairing of novel hemispherical image analysis of agricultural plots with the analysis of plant canopy movement in a systematic way enables differentiation of crop movement at the cultivar level. Though the analysis of plant movement from video data predates digital video, we consider our method generalizable to available camera technology and common field experimental designs for measuring movement as a plant phenotype for breeding or other biological purposes [20].

Demarcating individual crop rows from 360 video is novel with respect to high throughput image analysis of agricultural research plots, which is often performed using UAV captured images. Demarcating plots from a 360 image negates the need for stitching images captured by UAV mounted cameras, though at the cost of a smaller field coverage on a per image basis compared to multiple images stitched into one from a UAV [21]. In methodological studies of UAV image alignment of agricultural plots, regions of a field are identified through ground control points (GCP) with known GPS coordinates, which can be manually tagged in each image and then used to aid alignment along with shared spectral features in the images being stitched [22]. Plot misalignments induced by stitching error depend on the height of the UAV, spectral properties of the image, and number of GCPs. Among published studies, these stitching errors on average range from 1.5 to 4.5 cm at UAV heights of 30 m and 100 m respectively, and 8 cm to 13 cm depending on the spectral band analyzed and the number of GCPs used [23, 24]. Our average plot coordinate misalignment errors of 1.78 cm for inner reps (at 2.28 m from the camera track) and 8.74 cm (at 6.86 m from the camera track) for outer reps falls within the errors induced by stitching algorithms using GCPs. In theory, mounting the camera higher would decrease the error due to angle estimation errors by the plotfinder script (Additional file 1) at a given distance, as points would shift towards the center (pole) of the 360 image. While a high image spatial resolution is not critical for the measurement of plant movement from a time-varying color signal, decreasing this resolution will make subsequent analyses more sensitive to errors in camera system alignment or angle calculation. Despite alterations to camera system dimensions, the final errors will heavily depend on the panel being in alignment with the field design, as any misalignment will propagate at further distances even if the angle of the panel is accurately quantified by the plotfinder script. The plot demarcation method could be adapted to other cropping systems and field designs that desire a large field of view and the possibility for repeated imaging or videos of agricultural plots [19] with careful construction of the camera system.

The plot demarcation and plant movement quantification methods are well integrated via the use of the panels that enable normalization of color values in RGB video frames and identification of plots within the RCB field design. From this normalization and subsequent bandpass filtering of raw waveforms, we were able to compare parameters of plant movement across videos where different lighting conditions were present. Bandpass filtering also allowed for the removal of higher frequency (> 5 Hz) color changes due to camera vibrations induced at sufficiently high windspeeds on the camera system, leaving a signal of plant movement for analysis. The ability to treat parameters of plant movement such as natural frequency and amplitude as phenotypes that can be analyzed in common experimental designs such as the RCB represents a rigorous analytical framework for further testing models of plant wind interactions in the field. In the test of the plot demarcation and signal processing pipelines, we analyzed the RCB separately at five different dates. Lighting and wind conditions are expected to vary over different dates, and changes in lighting conditions (due to sun elevation or cloud cover) at the time of imaging will affect the absolute amplitude measurements as they are quantified based on the area under the bandpassed waveforms. However, a variety prone to larger amplitude movements will still be detected relative to those with lower amplitude movements when measured over a constant area of the plant canopy (929 cm^2^ in our case), even if the magnitude of the absolute amount of movement is less due to lighting conditions in a given video. Thus, a researcher employing this method over an entire growing season to investigate biological questions would be advised to employ a repeated measures ANOVA account for variation due to windspeed and lighting conditions among sampling dates in a final model of plant movement comparing varieties or crops. The RCB field design we used allowed us to statistically test the results from the video and signal processing pipelines by comparing relevant experimental effects and their interactions at seperate video dates. The presence of a significant effect of replicate position (inner vs outer) for natural frequency indicates that blocking the experiment with camera distance, as was done here, is critical in minimizing the effect of distance from the camera on mean natural frequency. Such effects on the variation in mean natural frequency are likely manifestations of errors in angle calculation identified in lower correlations between automatic and hand plot demarcation, indicating that there is more error in quantifying color changes at greater distance from the camera. This primarily stems from errors in calculating *Θ*_*off*_, resulting in a shift of the 929 cm^2^ region to another plot and capturing a color signal that is not representative of the intended plot canopy. While the plots captured in the hemispherical still frames occur at varying latitudes (viewing angles) in the images that are in theory affected by different lighting conditions, the predominant lack of a significant position effect on plant movement where the total amount of color change over time is directly quantified suggests that this issue is not as critical as improper angle calculation for accurate measurement of plant movement. That said, care should be taken to account for variation in lighting conditions at different viewing angles within hemispherical images for other applications if specific indices (i.e. ratios of spectral bands) are desired.

Mechanical estimates of plant movement, while useful for theoretical validations, are not as easily scaled to the level of field agricultural experiments compared to video based, colorimetric methods. Manually collected data on crop movement has been used to select for lodging resistance in cereals in the absence of naturally induced lodging, such as the ‘snapback’ trait in oats and other cereals [25]. The snapback trait consists of a researcher drawing a cereal row back, and rating (i.e. 1–10 scale) the strength and springiness of the stem [26, 27]. The low trait heritability of subjective snapback scores spurred the development of a more precise measure of stem displacement through hanging weights at a standard location along cultivar stems, thereby providing an estimate of torque that a stem can resist versus the torque that is applied (Coefficient of Lodging Resistance) [26]. Nonetheless, both snapback and coefficient of lodging resistance traits are hindered by subjective or cumbersome measurement. Free vibration tests, where a researcher pulls the main cereal stem back a set distance and manually times the subsequent oscillations, represents a standard field measurement technique for stem frequency for validating models of plant movement [6, 28]. Mechanical estimation of plant movement through accelerometer data, while increasingly feasible with compact sensors, poses difficulties in large field experiments such as the RCB design we used given difficulties in scaling materials and synchronizing data across all plots at any given point in time, notwithstanding possible mechanical interferences with plant movement [5]. Regardless of the method used to obtain plant movement data, any signal of wind induced plant movement will be highly intermittent given the inconsistent nature of wind speeds and direction at canopy level and the low frequency of plant oscillations. Both of these factors make signals of plant movement poor candidates for analysis via standard signal processing techniques such as the fourier transform. While errors might be induced due to physical properties of the camera system used, the ability to analyze multiple replicates of the same varieties provides the statistical power needed to differentiate cultivar differences in plant movement to provide insights for field investigations of plant-wind interactions.

The cultivar mean natural frequency (*ω*_*n*_) values estimated by our method fall within *ω*_*n*_ values either theoretically or empirically determined for cereal crops. Theoretical natural frequencies of a generic cereal crop range between 1.10 and 5.31 Hz, based on dimensionless parameters while empirical data on in-field wheat stems (cultivar ‘Mercia’) using a manual free vibration resulted in an average natural frequency of 0.91 Hz [7, 9]. Additional empirical data from free vibration tests in spring barley indicate an average natural frequency of 0.67 Hz and 0.60 Hz for the cultivars Golden promise and Optic, respectively [6]. While mean *ω*_*n*_ values calculated for the cultivars in our study were higher on average (1.37 Hz) than those reported for different cultivars of the same cereal crops, barley cultivars in our study generally had lower resonant frequencies than the wheat cultivars, a trend also apparent in [6]. Observations taken on the video date with the highest average wind speed and gusts were almost universally lower in mean *ω*_*n*_ values for cultivars and planting dates, which is to be expected as the wind moment increases thereby reducing the natural frequency by the square root of the increase in wind moment, all else equal [5] (Eq. 2) (Table 6). Considering its generalizability to different field applications, these video and signal processing methods represent good candidates for obtaining more information about the phenomenon of plant movement and its relationship to lodging resistance.

## Conclusions

We submit a novel video analysis pipeline that enables the automatic demarcation of agricultural plots from 360 video and a signal processing pipeline for analyzing color signals of plant movement in common field experimental designs. When coupled together, this method can be used to analyze video taken under varying wind speeds, allowing for the quantification of plant natural frequencies of movement under direct wind stress where typical high throughput phenotyping platforms (i.e. UAVs) are not suitable. When applied over a growing season, this method should amend itself to discerning trends in plant movement over time. These could include the physiological relationships between physiology, movement, and lodging, or even thigmotropic responses of plant tissues to plant movement [29]. In the broader goal of improving lodging resistance of cereal crops, the analysis of plant movement from 360 video will expand the quantifiable variation of this complex trait for use in plant improvement.

## Methods

### Camera, field, and naming conventions

#### Camera system

We designed and constructed an automatable camera track system to collect hemispherical video of single row research plots [19]. Briefly, the camera track system spanned 39.6 m at 2.7 m above ground level and deployed a 360fly 4k hemispherical video camera (360fly, Inc.). The camera tracking was repurposed industrial curtain tracking (AmCraft Manufacturing, Inc.) mounted on 3.65 m, 5.08 × 5.08 cm steel posts set 61 cm into concrete form tubes below the field surface. Movement of the camera along the track was accomplished by attaching the camera to a custom trolley linked to by a timing belt to a solar powered motor. Control of the motor was automated using a Raspberry Pi 3 Linux computer (Raspberry Pi Foundation). An example photo of the camera track is shown (Fig. 1). Importantly, 91 × 61 cm painted panels (panels) with a unique black, white, and red block design were centered underneath the camera track at regular intervals with the red square always oriented in the northwestern corner of the installed panel (Fig. 2a). The unique black and white block design served to uniquely mark the field location at each panel, while the red square served to orient the camera and apply necessary corrections to the geometric transformation of the 2D field into a 3D, hemispherical image.

#### Camera specifications

The 360fly 4k hemispherical video camera (referred to as 360 camera) recorded plant movement in the field. The 360 camera contained an 8 Elements Glass Ultra Fisheye Lens with an aperture of F2.5, effective focal length of 0.88 mm, and a minimum focus distance of 30 cm. The horizontal field of view was 360°, while the vertical field of view was 240°. Brightness was set to full brightness, while the aperture and contrast settings were set in the middle values for each. Videos were recorded at 24 frames per second with a per frame image size of 2880 × 2880 pixels. The 360fly ios app (V 2.0.0) was used to maintain consistent settings and initiate camera recordings while the camera was mounted on the track system, and the 360fly Director (V 0.10.4.0) software for Microsoft Windows 10 was used to download the videos from the camera and export them into.mp4 format.

#### Field design

The camera system imaged a randomized complete block (RCB) design. The design contained four cultivars of four different cereal grains (Table 1) for a total of 16 cultivars. Cereal cultivars were planted in single row plots (hereafter referred to as plots) of 3.04 m in length with 30 cm spacing between rows with a north–south orientation. A row of winter wheat separated each cereal plot used in the study. Each cultivar was replicated 8 times, with four replicates sufficiently close to the camera for analysis. Furthermore, this design was planted at four different planting dates (25 Apr, 5 May, 15 May, and 26 May 2017) in adjacent regions of the field. The camera system bisected the center of each planting date of the experimental design between the 4th and 5th replication, allowing video recording of different cultivars and growth stages at each video recording date. Average wind speed, gust, and direction data were obtained for each video date from the Minnesota DNR Mesonet station located on the research plots of the Minnesota Agricultural Experiment Station in St. Paul, MN (Table 2) [30].

#### Pre-analysis procedures

Prior to identifying plot regions within the 360 video and subsequent signal processing, we defined the field coordinate system and plot naming conventions. This allowed us to generalize the matlab scripts in the pipeline across different parts of the field RCB as indicated by the unique panels. The lodging RCB was numbered in a serpentine pattern beginning in the northeastern corner of the field, moving to the west, and then back to the east in the next replicate at each panel (see Additional file 3 for an illustration). We organized the plot naming and field coordinates according to this serpentine order. At each camera stopping point over a panel, 4 blocks were in view that contained between 22 and 28 plots in total. We created two files associated with each panel, one for plot names and the other for plot coordinates. For our field layout, the plot name files (Additional files 4, 5, 6, 7) were 22–28 lines depending on the panel it was associated with and contained the cultivar names associated with the serpentine numbered plots, beginning in the northeast corner of the analyzable field region. Each plot was named: *Cultivar_Crop_PlantingDateRep* (i.e. Gopher_oat_4A).

This allowed for regular expressions to search the names and append relevant plot data to the signal output from the videos. The plot coordinates file (Additional files 8, 9, 10, 11) contained the same number of rows as the plot names file for a given panel. The columns in the coordinates files contained the x, y, and z coordinates for each corner of a plot to represent the top of the canopy in 3D space. The first column contained plant heights (z values) for each plot. The remaining 8 columns contained the cartesian (x, y values) coordinates for each corner of a plot (SW, SE, NW, NE), with 2 columns (containing x, y distances) for each corner of the plot. These plot coordinates are hand-defined by the researcher and utilized in subsequent transformations to demarcate regions in the hemispherical image. We present distances in feet within the supplemental files given our field planting equipment, but ultimately these units are arbitrary. Plot coordinates were expressed from the center of the panel, with the x axis denoting the camera track path, and the y axis perpendicular to the camera track at the panel center point (origin). In our field setup, points north of the x axis had positive y values while those south had negative y values, and points east of the y axis had positive x values while those west had negative x values.

#### Video naming protocol

We set a standard file naming convention for videos (.mp4 files) so that the date information could be obtained throughout the video analysis and signal processing pipelines. We used the following file naming convention for videos: FLYmm_dd_yy_pn.mp4, with n being the panel number imaged in that video.

### Plotfinder analysis to demarcate agricultural plots in a 360 image

This portion of the methods section details the demarcation of agricultural plots from images taken using the 360 camera along with researcher defined field coordinates and plot names. The text below explains the processes coded in Additional file 1. Analysis was written in Matlab v2016b, and must be executed in Matlab v2016b or later.

#### Section 1.1: locate panel in the 360 video still frame (image)

The panel, identified by the red square in the northwest corner of each panel, provides a reference upon which all subsequent transformations of the field design are based. Masking the red square in the northwest corner of the panel provides a reference point for all measurements to conduct this transformation. The red square is masked by applying a threshold (with threshold values determined using the Matlab color thresholder application) to the 360 image in RGB color space (R: 251-255, G: 0-159, B: 0-255). Depending on ambient lighting conditions, these threshold values may need to be changed for individual videos to completely identify the red square. To ensure that the masked object using the threshold is the red square on the panel, square structuring elements of 4 pixels in size helped define the right angle edges of the red square, while all masked objects whose areas were < 5000 pixels (the area of the true red square is approximately 6000 pixels in the center of the image) were then unmasked, leaving only a masked image of the red square. While not experienced in this experiment, sunlight conditions that create an excessively saturated image might necessitate altering the minimum red square size or ensuring that the red square is adjacent to black panels instead of white to better define the location of the red square. With the red square masked from the original image, the left-most corner of the square serves as the reference point for all subsequent measurement (Fig. 2b). The leftmost corner of the red square is identified by finding the minimum column index of the image array where a non-zero element is present. Knowing the column index for the leftmost corner, the row index is found where the minimum row in the minimum column index is not zero. The column and row indices for the left most corner are then retained in memory as the script advances.

#### Section 1.2: identify direction and calculate angle of rotation

Estimating the direction and angle (in degrees) of rotation of the panel within the image allows the field design to be rotated to the correct orientation in the image. The column index of the leftmost corner of the red square is used to determine which direction the panel is facing in relative terms within the 360 image, as the camera axes might not be in line with the field axes when the camera is installed on the track system. The direction of rotation is determined by obtaining the RGB color values of the 40 pixels above and below the leftmost corner column (Fig. 2b). A mean red color value of > 245 will indicate the presence of a white square, while lower values will indicate that the ground is in view. Thus if the mean value of the indices below the left most column is > 245 while the mean above the left most column is < 245, the panel is rotated northeastward relative to the camera axes (Fig. 2a, b). Once the direction of rotation is known, the degrees rotated in that direction can be calculated from the remaining indices of the masked red square. In Fig. 2c representing a northeastern rotation of the panel in the image, the angle between the camera x axis and the field x axis (*Θ*_*off*_) is calculated using the relationship

3a $$\varTheta_{off} = - 90 - \tan\,{\text{d}}^{ - 1} \frac{{\overline{AB} }}{{\overline{BC} }}$$

While in Fig. 2d representing a southwestern rotation (as well as the northwest and southeastern rotations) of the panel in the image, the same angle is calculated

3b $$\varTheta_{off} = \tan\,{\text{d}}^{ - 1} \frac{{\overline{AB} }}{{\overline{BC} }}$$

With the points A,B,C representing the same triangulation (in row indices for AB and column indices for BC) of the panel offset angle in the image. The leftmost corner of the red square will not always represent the same point on the physical panel (Fig. 2c, d). Thus, the script will identify the indices of the relevant corner given a certain rotation condition.

#### Section 1.3: identify the panel in frame and incorporate field design

The researcher assigns the panel in frame a number that corresponds to those in the field coordinates or plot names files so that the correct versions of these files are used in subsequent transformations. The panel is identified based on user input at line 14 of the plotfinder script (Additional file 1). Once the panel is known, the corresponding plot names and plot coordinates files are read into the script from the working directory.

#### Section 1.4: rotate field design, transform field design to spherical coordinates

This section describes the rotation of the field design according to the direction of rotation and *Θ*_*off*_ value calculated in section 1.2. Following this rotation, the rotated 2D plot coordinates are transformed into spherical coordinates for use in identifying their location on a hemisphere. Each set of field (x, y) coordinates are extracted from the file into an *n* × *2* matrix, which is then transposed to a *2* *×* *n* matrix. All points in the *2* × *n* matrix are rotated clockwise around the origin by multiplying with a *2* × *2* rotation matrix. In the case of a northeastern rotation of the panel (Fig. 2c) and a *2* × *n* matrix representing the x and y coordinates of the southwestern corner points of the plots, the calculation is:

4a $$\left[ {\begin{array}{*{20}c} {{\text{cosd}}\varTheta_{off} } & {{\text{sind}}\varTheta_{off} } \\ { - {\text{sind}}\varTheta_{off} } & {{\text{cosd}}\varTheta_{off} } \\ \end{array} } \right]*\left[ {\begin{array}{*{20}c} {SW_{y1} } & {SW_{y2} } & {SW_{yn} \ldots } \\ {SW_{x1} } & {SW_{x2} } & {SW_{xn} \ldots } \\ \end{array} } \right] = \left[ {\begin{array}{*{20}c} {SW_{y1}^{\prime } } & {SW_{y2}^{\prime } } & {SW_{yn}^{\prime } \ldots } \\ {SW_{x1}^{\prime } } & {SW_{x2}^{\prime } } & {SW_{xn}^{\prime } \ldots } \\ \end{array} } \right]$$

For a southwestern rotation of the panel (Fig. 2d) in the image, 180° is added to theta offset in the rotation matrix:

4b $$\left[ {\begin{array}{*{20}c} {{\text{cosd}}(\varTheta_{off} + 180)} & {{\text{sind}}(\varTheta_{off} + 180)} \\ { - {\text{sind}}(\varTheta_{off} + 180)} & {{\text{cosd}}(\varTheta_{off} + 180)} \\ \end{array} } \right]*\left[ {\begin{array}{*{20}c} {SW_{y1} } & {SW_{y2} } & {SW_{yn} \ldots } \\ {SW_{x1} } & {SW_{x2} } & {SW_{xn} \ldots } \\ \end{array} } \right] = \left[ {\begin{array}{*{20}c} {SW_{y1}^{\prime } } & {SW_{y2}^{\prime } } & {SW_{yn}^{\prime } \ldots } \\ {SW_{x1}^{\prime } } & {SW_{x2}^{\prime } } & {SW_{xn}^{\prime } \ldots } \\ \end{array} } \right]$$

The rotation matrix for a northwestern rotation is the same as in Eq. 4a, while a southeastern rotation will have 90° added to *Θ*_*off*_ instead of the 180° as in Eq. 4b. In addition to adding 90° or 180° to *Θ*_*off*_, it is necessary to flip the sign of all field coordinate values in a southeastern or southwestern rotation condition. This is done by element wise multiplication of − 1 to all values within the coordinate matrices prior to multiplication by the rotation matrix.

Following rotation of the field coordinates in relation to the camera axes, the rotated field coordinates are transformed from the Cartesian (x, y) to spherical (latitude, longitude) coordinates. This is accomplished by two separate triangulations in three dimensional space (Fig. 3). The latitude angle (*φ*_*n*_) for a given coordinate *n* is calculated by taking the inverse sine of the ratio of plant height to the distance from the camera height to the *nth* point on the field:

5 $$\varphi_{n} = \sin\,{\text{d}}^{ - 1} \frac{{z_{n} }}{{\sqrt {x_{n}^{\prime } + y_{n}^{\prime } + z_{n} } }}$$

With the value of *φ*_*n*_ ranging from 0° to 90°. The longitude angle (*Θ*_*n*_) for a given coordinate is calculated by taking the inverse 4-quadrant tangent of the ratio of y to x distance to the rotated field coordinate:

6 $$\varTheta_{n} = \tan 2{\text{d}}^{ - 1} \frac{{y_{n}^{\prime } }}{{x_{n}^{\prime } }}$$

With the value of *Θ*_*n*_ ranging from − 180° to 180°. The *nth* plot coordinate is then represented in a new *n* × *2* matrix containing the latitude and longitude values for each coordinate in the rotated hemispherical image. These values are stored in plot order corresponding to that given in the plot coordinates and plot names files.

#### Section 1.5: obtain indexed pixel values as a function of spherical coordinates for each field design coordinate

Once plot coordinates are transformed from Cartesian to spherical coordinates, the latitude for a given point can then be represented in terms of the distance from the center of the image as a percentage of total pixels. This assumes that the image was taken directly above the panel so that the image center (pole) and the panel center are in alignment. The relationship between degrees latitude and number of pixels is non-linear: as latitudinal coordinates approach the equator (0°), fewer pixels are required to represent the change in latitude. Given the 240° field of view captured by the 360Fly 4K camera, the edge of the hemisphere does not represent the equator. Instead, the radius from the image center to the equator is assumed to be located at the 1440th row for frames taken at a 2880 × 2880 pixel resolution. Due to the field of view and other unknowns regarding the lens optics, we empirically determined the relationship between degrees latitude and the percentage of pixels in the image along the radius at the 1440th row. The percent distance (*D*_*n*_) in an image is thus a quadratic function of a point’s latitude

7 $$D_{n} = \varphi_{n}^{2} *10^{ - 7} - \varphi_{n} *5^{ - 3} + 0.3609$$

As the *1* × *n* matrix of *D*_*n*_ values represents the hypotenuse out to a given point based on the *n*th point’s latitude and longitude, this distance *D*_*n*_ for a point is used to estimate the distance (in percent of total indices) along the x and y axes within the image. The percentage of the image in terms of rows (*R*_*n*_) is calculated as

8a $$R_{n} = ~\frac{{D_{n} *\sin \;{\text{d}}\theta _{n} }}{{sin\;d\left( {90} \right)}}$$

And the percentage of the image in terms of columns (*C*_*n*_) is calculated as

8b $$C_{n} = \sqrt {D_{n}^{2} - R_{n}^{2} }$$

The resulting *1* × *n* matrices of *R*_*n*_ and *C*_*n*_ values are concatenated into a *2* × *n* matrix of the same dimensions as the initial coordinate input for a given set of points. Expressing distances as a percent of the image size from the image center (radius), they represent the percent distance in rows and columns to a given point from the field design on the hemispherical image.

#### Section 1.6: create correction matrix for points in field design for applying correct index sign

Though the row and column indices are expressed as percentages of the radius of the hemispherical image, the calculations in section 1.5 do not assign the correct sign to these indices to indicate their location in regards to the image center. The *R*_*n*_ and *C*_*n*_ values calculated above are expressed in percent distance from the image center without the correct sign indicating where the points fall with respect to the image center. Longitude angles for the *nth* point are used to assign the appropriate sign to the *R*_*n*_ and *C*_*n*_ values. For rows, if the longitude angle for the *nth* point is between 0 and 180°, *R*_*n*_ will be negative while if the longitude angle is between 0 and − 180°, *R*_*n*_ will be positive. For columns, if the longitude angle for the *n*th point is between 90 and − 90°, *C*_*n*_ will be positive while if the longitude angle is between 90 and 180° or − 180 and − 90°, *C*_*n*_ will be negative. The correction matrix will have dimensions of *n* × *2*, with the first column containing the row distance as a percentage of image size, the second column containing the column distance as a percentage of image size, and *n* rows for the number of points in the field design.

#### Section 1.7: apply the correction matrix to the index values

The correction matrix indicating the correct sign for the row, column index percentages is applied, with the index percentages converted to actual pixel indices based on the image size, and then added to the image center row, column index to obtain the final transformation of the field coordinates to their location in the hemispherical image. The correction matrix for each coordinate is multiplied element-wise by the matrix of row and column distance percentages

9 $$\left[ {\begin{array}{*{20}c} { - 1} & 1 \\ 1 & { - 1} \\ \vdots & \vdots \\ \end{array} } \right]*\left[ {\begin{array}{*{20}c} {R_{1} } & {C_{1} } \\ {R_{2} } & {C_{2} } \\ {R_{n} } & {C_{n} } \\ \end{array} } \right] = \left[ {\begin{array}{*{20}c} { - R_{1} } & {C_{1} } \\ {R_{2} } & { - C_{2} } \\ {R_{n} } & {C_{n} } \\ \end{array} } \right]$$

This resulting matrix contains the *R*_*n*_ and *C*_*n*_ values whose signs reflect the correct orientation with respect to the image center. The *R*_*n*_ and *C*_*n*_ distance percentages are then multiplied element-wise by the image size (2880 pixels) express these values as image pixels. Since these values now represent the number of pixels to the *nth* point along the image y and x axes from the center of the image, half the value of the image size (1440 pixels) is added to every element within this matrix. The resulting array contains the indexed pixel value for the row and column in the image where a plot coordinate is located.

#### Section 1.8: crop plots based on these rotated coordinates, export images and cropping functions

With each of the four corners of a plot represented in terms of indexed rows and columns pixels in the image, demarcation of the original image can begin. Each row in the resulting pixel arrays for plot corners (NW, NE, SE, SW) represents a unique plot in the field design that corresponds with the order in the original plot coordinates and plot names files. The *nth* row of each of the four arrays containing row and column pixels are used to demarcate a polygon cropping region on the original image with the plot name appended to the filename according to the *nth* row of the plot names file. The script will produce the same number of images as the number of rows in the input plot coordinates and plot names files. Additionally, a cropping function is generated by the script that can demarcate the plot region across frames in a video. Examples of the resulting cropped plots in different rotation conditions and region of interest sizes are shown (Fig. 4a–d). The correctness of the plot demarcation can be visually assessed by the user though comparing the cropped image of a plot with the entire field captured in one of the uncropped frames.

#### Plotfinder accuracy

We assessed the plotfinder accuracy by comparing the differences in feet on the ground generated by automatic angle detection and manual angle measurement. We ran the still frames from each video through the plot finder script to calculate *Θ*_*off*_ and obtain the rotated coordinates for each corner of the demarcated 929 cm^2^ area. We then manually calculated the angle of the red square by measuring the distance manually using the line tool in Powerpoint (Office 365), which gives the height and width that the line travels in the image and used those values to calculate *Θ*_*off*_ manually (Eq. 3a–b). This value of *Θ*_*off*_ was then used to recalculate the x and y coordinates of each plot corner. The average x, y values of the outer and inner reps were then grouped and averaged, with the x and y differences calculated before a final averaging of the x and y differences for the outer and inner reps.

To evaluate the automatic plotfinder script demarcation error on quantifying color changes indicative of plant movement, we manually outlined cropping function demarcating 929 cm^2^ regions (as in Fig. 4c, d) of the 98 plots at each of the 5 video dates using the image segmenter app built into Matlab v2016b. We used this manual demarcation to compare with the automatically generated cropping function using the plotfinder script and plot names, plot coordinates files that denoted 929cm2 regions in the field. Both the manual and automatically generated cropping functions were applied over each frame to calculate the average normalized red color value in the demarcated region. The average normalized red values from the manual and automatically generated cropping functions were then correlated over all frames and planting dates for each video date. A linear model with one term indicating whether the position of the rep (inner or outer) was used to explain the correlation for each video date (across planting dates, plots). An LSD test with Bonferroni correction in the R package agricolae was then used to assess whether the differences in mean correlation coefficients between the inner and outer reps was significant.

### Movement Analysis using the plotfinder generated cropping functions

The second half of the method first uses two matlab scripts (Additional files 12, 13) written in MATLAB v2016b followed by two R scripts (Additional files 14, 15) written in R v3.4.1.

#### Section 2.1: obtaining raw waveform data on plant movement

We apply our plot demarcation method to a novel strategy for quantifying plant row canopy movement by analyzing the change in normalized red color values averaged within the demarcated plots of the RGB 360 4k video. While reflectance of plant tissue is low in the red band of visible light due to absorption by chlorophyll, the soil between rows reflects wavelengths in this part of the visible spectrum back to the camera [31]. Since the stem oscillations are captured against ground surfaces or large gaps in foliage in this field layout, the red channel of the RGB 360 4k video should be best suited to detecting movement of a cereal row canopy within a fixed region across all frames. These specific regions correspond to a 929 cm^2^ region at the height of the plant canopy with one edge along the center of the row. These functions were called by a script that was unique to each panel (Additional file 12). This script incorporated the plotfinder generated cropping functions specified for each plot, and calculated the average red value at each frame for every plot. The average red value for each plot was then normalized to the red square on the panel at that frame in the video, so that color values could be compared across different lighting conditions within a video and across videos. Each waveform script produced a resulting array with the number of rows equaling the number of frames in the video, and the number of columns equaling the number of plots. The arrays for each video are exported as a text file with the same file name as the video analyzed. These steps are then repeated for all the videos. This enables collection of the waveforms in plant row canopies to be quantified and expressed in normalized red color units, as shown in Fig. 5a.

#### Section 2.2: organizing raw waveform data on plant movement for analysis

We quantify the amount of canopy movement within each of these demarcated regions of the plot using a time domain signal processing approach (Additional file 13). First, we utilized a bandpass filter on each plot’s waveform to filter out low frequency changes in red values due to light changes from passing clouds during a video. The bandpass filter centers each waveform at 0, enabling comparisons of canopy movement across different lighting conditions (within and among videos) and across different cereal cultivars, which have different mean red color values. The equiripple bandpass filter implemented in the MATLAB script contained the following filtering parameters as expressed in percentage of analyzable frequencies (0–12 Hz in this case given the 24 fps recording rate). Filtering began with an Fstop1 of 3%, Fpass1 of 4%, and ended with an Fpass2 of 40%, Fstop2 of 50% to eliminate high frequency (> 0.5 Hz) noise due to camera vibrations induced by high winds. The attenuation of the passed versus filtered signal was 100 dB, while the Ap value within the passed signal was 5 dB. This filter was applied to each column in input text file of raw plot waveforms from a video.

Following bandpass filtering of each waveform, we obtained the amplitude (in normalized red color units) and time location (in frames) of each local maxima of the waveform color signal with at least a 0.5% red change prominence. Next, we calculated the distance (in frames) between peaks, skipping the first peak. A visualization of the peak identification process is presented in Fig. 5b. These distances were used to estimate the frequency between peaks, as the distance in frames was divided by the frame rate (24 fps). Finally, we summed the total (absolute) area under the curve that lies between peaks to estimate the amplitude change in the waveform. The data was then written to a text file for each plot containing one column listing the frequency of a given cycle, and the absolute area encompassed by that cycle. For each video, a subdirectory was written that contained the individual files for each plot.

This frequency and area data was run through an R script (Additional file 14) to organize it for analysis. The user navigates to the directory containing all video dates for one panel. The r script then goes through each subdirectory to read in the individual plot files containing the frequency and area data. Information including cultivar, replicate, video date, and planting date are obtained from the standard naming protocol outlined above. For each file, the pass range (between 0.5 and 4.9 Hz) is used to calculate a mean, median, and standard deviation of the frequency distribution for each plot (Fig. 5c). The areas in the plot file are then binned according to their corresponding frequencies into 0.2 Hz bins from 0.5 to 4.7 Hz. Each plot file is condensed to one line containing these values, with the resulting dataframe for one video date containing a row for each plot. A position vector indicating whether a plot is near or far from the camera track is added last, with the numbers corresponding to the file alphanumeric order in the subdirectories as opposed to how they are physically laid out in the serpentine pattern. Finally, all of the dataframes for each video date are concatenated into a single dataframe for each panel. Dataframes for all panels are then concatenated into a final dataframe for the statistical analysis of plant movement.

#### Section 2.3: statistical analysis of plant row movement

Plant row movement was modeled using the components of a randomized complete block design (Additional file 15). We analyzed two response variables of plant movement: the mean frequency (*ω*_*n*_) of the peak distribution for each row in a video date, as well as the total (absolute) area between peaks in the 1.1–1.3 Hz frequency band. Each response variable was modeled using the following linear model:

10 $$y_{ijkl} = \beta_{0} 1 + \beta_{1} x_{i1} + \beta_{2} x_{i2} + \beta_{3} x_{i\left( j \right)3} + \beta_{4} \left( {x_{i1} * x_{i2} } \right)_{k} + \beta_{5} \left( {x_{i1} * x_{i\left( j \right)3} } \right)_{l} + \varepsilon_{ijkl}$$

With *ß*_*1*_ representing the cultivar effect, *ß*_*2*_ the planting date effect, *ß*_*3*_ the effect of a replicate in the *jth* position (inner or outer) nested within the *ith* cultivar, *ß*_*4*_ the cultivar by planting date interaction effect, and *ß*_*5*_ the cultivar by *jth* position nested within the *ith* planting date interaction effect. The ANOVA for estimating the significance of each effect was conducted using the expected mean squares and F values presented in the table below (Table 11).

**Table 11:** Expected mean squares and F test calculations for parameters in Eq. 10

Source of variation	Row effect	Expected mean squares	F value
Cultivar	Κ_C_^2^	σ_e_^2^ + σ_CxP(PD)_^2^ + Κ_C_^2^	(σ_CxP(PD)_^2^ + Κ_C_^2^)/σ_CxP(PD)_^2^
PlantingDate	Κ_PD_^2^	σ_e_^2^ + σ_P(PD)_^2^ + Κ_PD_^2^	(σ_P(PD)_^2^ + Κ_PD_^2^)/σ_P(PD)_^2^
Pos (PlantingDate)	σ_P(PD)_^2^	σ_e_^2^ + σ_P(PD)_^2^	σ_P(PD)_^2^/σ_e_^2^
Cultivar * PlantingDate	Κ_CPD_^2^	σ_e_^2^ + σ_CxP(PD)_^2^ + Κ_PC_^2^	(σ_CxP(PD)_^2^ + Κ_CPD_^2^)/σ_PxR(C)_^2^
Cultivar * Pos (PlantingDate)	σ_CxP(PD)_^2^	σ_e_^2^ + σ_CxP(PD)_^2^	σ_CxP(PD)_^2^/σ_e_^2^
Residuals	σ_e_^2^	σ_e_^2^	

The F values were tested according to the relevant degrees of freedom for each mean square component in the ratio using the probability density function for an F distribution in R. For all of these linear effects, an LSD test with a false discovery rate correction in the R package agricolae was used to quantify the mean value for each level within the response variable and assess significant differences in either mean frequency or total area at alpha = 0.05 [32]. The ANOVA and LSD tests were run separately on movement data obtained at each of the five video dates (Table 2).

## Additional files

**Additional file 1.** Plotfinder_published. Matlab script to demarcate user-defined regions of a field from hemispherical video

**Additional file 2.** FLY07_12_17_p2. MP4 video file from 360Fly 4k camera, taken 12 Jul 2017 at planting date 2. Used as sample video for published scripts

**Additional file 3.** Fieldmap_readme. Field design at the panel in planting date 2. Corresponds to analyzable plots in Additional file 2.

**Additional file 4.** FieldPlotnames7. Field plot names file for 25 Apr 2017 planting date

**Additional file 5.** FieldPlotnames5. Field plot names file for 5 May 2017 planting date

**Additional file 6.** FieldPlotnames3. Field plot names file for 15 May 2017 planting date

**Additional file 7.** FieldPlotnames1. Field plot names file for 25 May 2017 planting date

**Additional file 8.** FieldCoordinates7. Field Coordinates file for 25 Apr 2017 planting date

**Additional file 9.** FieldCoordinates5. Field Coordinates file for 5 May 2017 planting date

**Additional file 10.** FieldCoordinates3. Field Coordinates file for 15 May 2017 planting date

**Additional file 11.** FieldCoordinates1. Field Coordinates file for 25 May 2017 planting date

**Additional file 12.** FLY07_12_17_p2. Matlab script to obtain normalized waveforms from sample video (Additional file 2)

**Additional file 13.** BandpassParse_p2. Matlab script to filter and parse waveforms from Additional file 12.

**Additional file 14.** Stationary_organization_published. R script to organize the frequency, amplitude, and plot data from Additional file 13 into an statistically analyzable format

**Additional file 15.** Stationary_analysis_published. R script to analyze the data from Additional file 14.

## Data Availability

The datasets generated and analyzed during the current study are included within the article and additional files, which are available online. The sample video (Additional file 2) on which the analyses can be run is located at 10.13020/D6BT49. All other additional files and data are located in three Github repositories: the PlantMovementReadme repository (https://github.com/Hortus/PlantMovementReadme), the Plotfinder repository (https://github.com/Hortus/Plotfinder) and the MovementSignalProcessing repository (https://github.com/Hortus/MovementSignalProcessing).

## Abbreviations

*C*_*n*_: percent of image size along image y axis from image center to the nth point
*dB*: decibel
*D*_*n*_: distance to the nth point as mapped on the image (as a percentage of image size)
*fps*: frames per second
*GCP*: ground control point
*Hz*: hertz
*m*_*p*_: stem mass
*M*_*w*_: wind moment (height * force)
*n*: the nth coordinate point in a field design
*RCB*: randomized complete block
*R*_*n*_: percent of image size along image x axis from image center to the nth point
$SW_{xn} ,SW_{xn}^{\prime }$: field design x cartesian coordinates to the nth southwest corner, field design x cartesian coordinates to rotated nth southwest corner
$SW_{yn} ,SW_{yn}^{\prime }$: field design y cartesian coordinates to the nth southwest corner, field design y cartesian coordinates to rotated nth southwest corner
*UAV*: unmanned aerial vehicle
*x*_*n*_: field design x cartesian coordinates to the nth point
*y*_*n*_: field design y cartesian coordinates to the nth point
*z*_*n*_: field design z coordinates from the height of the canopy at the nth point to the height of the camera
*ζ*_*a*_: aerodynamic loading coefficient of plant stem
*ζ*_*s*_: structural loading coefficient of plant stem
*θ*: stem angle
$$\dot{\theta }$$: stem velocity
$$\ddot{\theta }$$: stem acceleration
*Θ*_*n*_: longitude angle of the nth point
*Θ*_*off*_: rotation angle of field design relative to camera axes
*φ*_*n*_: latitude angle of the nth point
*ω*_*n*_: natural frequency of a plant stem or cultivar row
